# Genetic aspects of congenital nephrotic syndrome: a consensus statement from the ERKNet–ESPN inherited glomerulopathy working group

**DOI:** 10.1038/s41431-020-0642-8

**Published:** 2020-05-28

**Authors:** Beata Stefania Lipska-Ziętkiewicz, Fatih Ozaltin, Tuula Hölttä, Detlef Bockenhauer, Sandra Bérody, Elena Levtchenko, Marina Vivarelli, Hazel Webb, Dieter Haffner, Franz Schaefer, Olivia Boyer

**Affiliations:** 1 Clinical Genetics Unit, Department of Biology and Medical Genetics, Medical University of Gdańsk, Gdańsk, Poland; 2 Centre for Rare Diseases, Medical University of Gdańsk, Gdańsk, Poland; 3 Department of Pediatric Nephrology and Nephrogenetics Laboratory, Hacettepe University Faculty of Medicine, Ankara, Turkey; 4 Department of Pediatric Nephrology and Transplantation, The New Children’s Hospital, HUS Helsinki University Hospital, Helsinki, Finland; 5 UCL Department of Renal Medicine and Renal Unit, Great Ormond Street Hospital for Children NHS Foundation Trust, London, UK; 6 Department of Pediatric Nephrology, Reference Center for Hereditary Kidney Diseases (MARHEA), Necker Hospital, APHP, 75015 Paris, France; 7 Division of Pediatric Nephrology, Department of Pediatrics, University Hospitals Leuven; Department of Development & Regeneration, University of Leuven, Leuven, Belgium; 8 Division of Nephrology and Dialysis, Department of Pediatric Subspecialties, Bambino Gesù Pediatric Hospital and Research Center, Rome, Italy; 9 Department of Pediatric Kidney, Liver and Metabolic Diseases, Hannover Medical School Children’s Hospital, Hannover, Germany; 10 Center for Congenital Kidney Diseases, Center for Rare Diseases, Hannover Medical School, Hannover, Germany; 11 Division of Pediatric Nephrology, Center for Pediatrics and Adolescent Medicine, Heidelberg, Germany; 12 Laboratory of Hereditary Kidney Diseases, Imagine Institute, INSERM, Paris Descartes University, U1163, Paris, France

**Keywords:** Focal segmental glomerulosclerosis, Genetic testing

## Abstract

Congenital nephrotic syndrome (CNS) is a heterogeneous group of disorders presenting with massive proteinuria within the first 3 months of life almost inevitably leading to end-stage kidney disease. The Work Group for the European Reference Network for Kidney Diseases (ERKNet) and the European Society for Pediatric Nephrology (ESPN) has developed consensus statement on genetic aspects of CNS diagnosis and management. The presented expert opinion recommends genetic diagnostics as the key diagnostic test to be ordered already during the initial evaluation of the patient, discusses which phenotyping workup should be performed and presents known genotype–phenotype correlations.

## Introduction

Congenital nephrotic syndrome (CNS) is a heterogeneous group of disorders presenting in utero or during the first 3 months of life with marked edema and massive proteinuria [1]. In the vast majority of cases, CNS is a primary glomerular disorder due to genetic defects; occasionally it can however be caused by congenital infections or alloimmune maternal disease [2].

Originally, the disorder has been referred to as the Finnish-type nephrotic syndrome due to its high incidence in Finland (1:8.000 live births), with two *NPHS1* founder mutations (i.e., Fin-major and Fin-minor) in most cases [3–5]. However, over the years several other genes have been implicated in the CNS etiology when mutated, both in isolated cases or in the the less common syndromic forms of the disease [6–8].

*NPHS1* (encoding nephrin) or *NPHS2* (encoding podocin) biallelic pathogenic variants are the most common causes of the disease in patients of both European and other descents. Among syndromic forms, *WT1*-associated glomerulopathy is the most frequent, followed by Pierson syndrome caused by *LAMB2* biallelic pathogenic variants [6–14]. The exact data on the prevalence of CNS in various populations are limited. With the advent of massive-parallel sequencing, rapid-WES usage in daily clinical practice in particular, it is anticipated that the information on the associated mutational spectrum and the incidence of various types of hereditary CNS will be soon achieved. Public lists of disease-causing variants are available online (www.ncbi.nlm.nih.gov/clinvar/; www.lovd.nl; www.hgmd.cf.ac.uk). Also, online case consultations, including discussion of variants in question, are available via the European Rare Kidney Disease Reference Network (http://www.cpms.erknet.org).

CNS management is very challenging due to high morbidity and mortality rates [15]. Patients with CNS are prone to severe complications related to hypoproteinemia such as recurrent infection, thrombosis, and impaired growth and most progress to end-stage kidney disease (ESKD) within a few years [1, 5, 16].

In 2018, the European Reference Network for Kidney Diseases (ERKNet) and the European Society for Pediatric Nephrology (ESPN) have jointly established a Work Group to develop recommendations for clinical diagnostics, management and treatment of CNS. This resulted in clinical recommendations for CNS (Boyer et al. in prep.) and the present consensus statement on genetic aspects of CNS diagnosis and management.

Because evidence is frequently missing or inadequate in CNS management, this article is an expert opinion paper rather than a clinical practice guideline. Detailed methodology, including formulation of PICO (Patient or Population covered, *I*ntervention, Comparator, Outcome) questions, approaches to literature search and three-step revision process is presented in the parallel clinical paper (Boyer et al. in prep.).

**Content list—questions addressed in the presented recommendations:**

1. *Which phenotyping workup should be performed?*
2. *What is the preferred time-point for genetic diagnostics?*
3. *What is the appropriate genetic testing approach?*
4. *Is there a role for karyotyping?*
5. *What kind of samples are needed for genetic testing?*
6. *Is there a role for prenatal diagnosis/genetic counseling?*
7. *What are the phenotype/genotype correlations?*
8. *How to manage syndromic forms?*
9. *What is the inheritance pattern of a hereditary CNS?*
10. *Parents as kidney donators.*

1. *Which phenotyping workup should be performed?*

In addition to standard clinical care phenotypic workup in infants with CNS (Boyer et al. in prep.) we recommend additional evaluation with the aim of identifying extrarenal signs and symptoms suggestive of an underlying genetic disease.

Clinical examination should be performed to identify the possible extrarenal manifestations of a hereditary form of CNS. Table 1 summarizes possible phenotypic manifestations of syndromic forms of CNS.

**Table 1:** Possible phenotypic manifestations of syndromic forms of CNS.

	Phenotypic feature	HPO code	Gene(s) associated with feature
General	Intrauterine growth retardation (IUGR)	HP:0001511	*WDR4, WDR73, LAGE3, OSGEP, TP53RK, TPRKB, NUP107, NUP133*
Head and neck			
*Dysmorphic features*	–Microcephaly,	HP:0000252	*WDR4, WDR73, LAGE3, OSGEP, TP53RK, TPRKB, NUP107, NUP133, SGPL1*
	–Sloping forehead,	HP:0000340	*WDR4, WDR73, LAGE3, OSGEP, TP53RK, TPRKB, NUP107, NUP133*
	–Flat occiput	HP:0005469	*WDR4, WDR73, LAGE3, OSGEP, TP53RK, TPRKB, NUP107, NUP133*
	–Small midface	HP:0011800	*WDR4, WDR73, LAGE3, OSGEP, TP53RK, TPRKB, NUP107, NUP133*
	–Microphthalmia	HP:0000568	*WDR4, WDR73, LAGE3, OSGEP, TP53RK, TPRKB, NUP107, NUP133*
	–Hypertelorism	HP:0000316	*WDR4, WDR73, LAGE3, OSGEP, TP53RK, TPRKB, NUP107, NUP133*
	–Epicanthal folds	HP:0000286	*WDR4, WDR73, LAGE3, OSGEP, TP53RK, TPRKB, NUP107, NUP133*
	–Ptosis	HP:0000508	*WDR4, WDR73, LAGE3, OSGEP, TP53RK, TPRKB, NUP107, NUP133, SGPL1*
	–Hypoplasia of the ear cartilage	HP:0100720	*WDR4, WDR73, LAGE3, OSGEP, TP53RK, TPRKB, NUP107, NUP133*
	–Microganthia	HP:0000347	*WDR4, WDR73, LAGE3, OSGEP, TP53RK, TPRKB, NUP107, NUP133*
*Vision*	–Nonreactive, fixed narrowing of the pupil ('microcoria')	HP:0025492	*LAMB2*
	–Aplasia or atrophy of the dilatator pupillae muscle	HP:0007686	*LAMB2*
	–Hypoplasia of the iris	HP:0007676	*WDR4, WDR73, LAGE3, OSGEP, TP53RK, TPRKB, NUP107, NUP133, LAMB2*
	–Hypoplasia of the ciliary body	HP:0007774	*LAMB2*
	–Lenticonus posterior	HP:0011502	*LAMB2*
	–Corneal opacities	HP:0007957	*WDR4, WDR73, LAGE3, OSGEP, TP53RK, TPRKB, NUP107, NUP133*
	–Cataracts	HP:0000518	*WDR4, WDR73, LAGE3, OSGEP, TP53RK, TPRKB, NUP107, NUP133*
	–Strabismus	HP:0000486	*WDR4, WDR73, LAGE3, OSGEP, TP53RK, TPRKB, NUP107, NUP133, SGPL1*
	–Nystagmus	HP:0000639	*COQ2, WDR4, WDR73, LAGE3, OSGEP, TP53RK, TPRKB, NUP107, NUP133*
	–Retinitis pigmentosa	HP:0000510, HP:0000547	*COQ2*
	–Optic atrophy	HP:0000648	*WDR4, WDR73, LAGE3, OSGEP, TP53RK, TPRKB, NUP107, NUP133*
	–Cortical visual impairment	HP:0100704	*PDSS2*
	–Vision loss (blindness)	HP:0000618, HP:0000572, HP:0000505	*COQ2, LAMB2*
*Hearing*	–Deafness, sensorineural	HP:0000407	*COQ2, COQ6, SGPL1*
Neurologic	–Global developmental delay	HP:0001263	*COQ2, COQ6, WDR4, WDR73, LAGE3, OSGEP, TP53RK, TPRKB, NUP107, NUP133, LAMB2, PDSS2 SGPL1*
*Central nervous system*	–Cognitive impairment	HP:0100543	*COQ2, COQ6, WDR4, WDR73, LAGE3, OSGEP, TP53RK, TPRKB, NUP107, NUP133, LAMB2, PDSS2*
	–Developmental regression	HP:0002376	*SGPL1*
	–Cognitive decline	HP:0001268	*SGPL1*
	–Impaired speech	HP:0000750	*WDR4, WDR73, LAGE3, OSGEP, TP53RK, TPRKB, NUP107, NUP133, SGPL1*
	–Hypotonia	HP:0001290,	*LAMB2, SGPL1*
		HP:0001252	
	–Hypotonia, neonatal	HP:0001319, HP:0008935	*PDSS2*
	–Seizures	HP:0001250	*CRB2, COQ2, COQ6, WDR4, WDR73, LAGE3, OSGEP, TP53RK, TPRKB, NUP107, NUP133, PDSS2, SGPL1*
	–Status epilepticus	HP:0002133	*PDSS2*
	–Encephalopathy	HP:0001298	*COQ2*
	–Hydrocephaly	HP:0000238	*CRB2*
	–Ventriculomegaly	HP:0002119	*CRB2*
	–Focal hyperplasia of the choroid plexus	HP:0007376	*CRB2*
	–Gray matter heterotopia	HP:0002282	*CRB2*
	–Hypotonia, axial	HP:0008936	*WDR4, WDR73, LAGE3, OSGEP, TP53RK, TPRKB, NUP107, NUP133*
	–Spastic quadriplegia	HP:0002510	*WDR4, WDR73, LAGE3, OSGEP, TP53RK, TPRKB, NUP107, NUP133*
	–Ataxia	HP:0001251, HP:0010867	*COQ2, WDR4, WDR73, LAGE3, OSGEP, TP53RK, TPRKB, NUP107, NUP133, SGPL1*
	–Dystonia	HP:0001332	*WDR4, WDR73, LAGE3, OSGEP, TP53RK, TPRKB, NUP107, NUP133*
	–Hyperreflexia	HP:0001347	*WDR4, WDR73, LAGE3, OSGEP, TP53RK, TPRKB, NUP107, NUP133*
	–Dilated ventricles	HP:0002119	*WDR4, WDR73, LAGE3, OSGEP, TP53RK, TPRKB, NUP107, NUP133*
	–Cerebellar atrophy	HP:0001272	*WDR4, WDR73, LAGE3, OSGEP, TP53RK, TPRKB, NUP107, NUP133*
	–Thin corpus callosum	HP:0002079	*WDR4, WDR73, LAGE3, OSGEP, TP53RK, TPRKB, NUP107, NUP133, COQ2*
	–Cerebral atrophy	HP:0002059	*WDR4, WDR73, LAGE3, OSGEP, TP53RK, TPRKB, NUP107, NUP133*
	–Dandy–Walker malformation	HP:0001305	*WDR4, WDR73, LAGE3, OSGEP, TP53RK, TPRKB, NUP107, NUP133*
	–Small brainstem	HP:0002365	*WDR4, WDR73, LAGE3, OSGEP, TP53RK, TPRKB, NUP107, NUP133*
	–Pachygyria abnormal gyri/abnormal sulci	HP:0001302	*WDR4, WDR73, LAGE3, OSGEP, TP53RK, TPRKB, NUP107, NUP133*
*Peripheral nervous system*	–Areflexia	HP:0001284	LAMB2
	–Peripheral neuropathy	HP:0001271, HP:0009830, HP:0000759	*SGPL1*
Chest			
*Cardiovascular*	–Ventricular septal defect	HP:0001629	*CRB2, NPHS2*
	–Hypertrophic cardiomyopathy	HP:0001639	*COQ2*
*Diaphragm*			
	–Diaphragmatic hernia	HP:0000776	*WT1*
Abdomen			
*Gastrointestinal*	–Hiatal hernia	HP:0002036	*WDR4, WDR73, LAGE3, OSGEP, TP53RK, TPRKB, NUP107, NUP133*
	–Feeding difficulties in infancy	HP:0008872	*PDSS2*,*WDR4, WDR73, LAGE3, OSGEP, TP53RK, TPRKB, NUP107, NUP133, SGPL1*
*Liver*			
	–Liver failure	HP:0001399	*COQ2*
Genitourinary			
*Kidneys*	–Urinary track malformations	HP:0000079, HP:0000119	*WT1*
	–Nephroblastoma (Wilms tumor)	HP:0002667	*WT1*
	–Hyperechogenic kidneys	HP:0004719	*CRB2*
	–Renal corticomedullary cysts	HP:0000108	*CRB2*
*Genitalia*	–Male-to-female sex reversal	HP:0000037	*WT1*
	–Ambiguous genitalia	HP:0000062	*WT1*
	–Micropenis	HP:0000054	*SGPL1, WT1*
	–Cryptorchidism	HP:0000028	*SGPL1, WT1*
	–Gonadal dysgenesis	HP:0000133	*WT1*
	–Testicular and ovarian tissue present	HP:0010459	*WT1*
	–Gonadal tissue inappropriate for external genitalia or chromosomal sex	HP:0003248	*WT1*
	–Gonadoblastoma	HP:0000150	*WT1*
Muscle/skeletal	–Joint contractures	HP:0001371	*WDR4, WDR73, LAGE3, OSGEP, TP53RK, TPRKB, NUP107, NUP133*
	–Muscle weakness, progressive	HP:0003323	*COQ2*
	–Ragged red fibers	HP:0003200	*COQ2*
*Hands*	–Clenched hands	HP:0001188	*WDR4, WDR73, LAGE3, OSGEP, TP53RK, TPRKB, NUP107, NUP133*
	–Camptodactyly	HP:0012385	*WDR4, WDR73, LAGE3, OSGEP, TP53RK, TPRKB, NUP107, NUP133*
	–Postaxial polydactyly	HP:0100259	*CRB2*
*Feet*	–Pes cavus	HP:0001761	*WDR4, WDR73, LAGE3, OSGEP, TP53RK, TPRKB, NUP107, NUP133*
	–Talipes equinovarus	HP:0001762	*WDR4, WDR73, LAGE3, OSGEP, TP53RK, TPRKB, NUP107, NUP133*
Skin	–Ichthyosis	HP:0008064	*SGPL1*
	–Hyperpigmentation	HP:0000953	*SGPL1*
	–Hypopigmentation	HP:0001010	*WDR4, WDR73, LAGE3, OSGEP, TP53RK, TPRKB, NUP107, NUP133*
	–Hypoplastic nails	HP:0001792	*WDR4, WDR73, LAGE3, OSGEP, TP53RK, TPRKB, NUP107, NUP133*
Endocrine	–Adrenal insufficiency	HP:0008207, HP:0000846	*SGPL1*
	–Adrenal calcifications	HP:0010512	*SGPL1*
	–Glucocorticoid deficiency	HP:0008163	*SGPL1*
	–Hypoglycemia	HP:0001943	*SGPL1*
	–Hypothyroidism	HP:0000821	*SGPL1*
	–Hypogonadism	HP:0000135	*SGPL1*
	–Primary amenorrhea	HP:0000786	*WT1*
	–Infertility (male)	HP:0003251	*WT1*
	–Diabetes mellitus	HP:0000819	*COQ2*
Laboratory	–Lymphopenia	HP:0001888	*SGPL1*
	–Lactic acidemia	HP:0003128	*COQ2, PDSS2*
	–Pyruvic acidemia	HP:0003542	*PDSS2*
	–Increased serum creatine kinase	HP:0003236	*COQ2, PDSS2*
	–Anemia	HP:0001903	*COQ2*
	–Pancytopenia	HP:0001876	*COQ2*

Particular attention should be paid to neurological examination, including brain imaging in selected cases. Abnormal cerebral gyration or cerebellar atrophy in Galloway–Mowat syndrome (GAMOS) [17–19]; cerebral and cerebellar atrophy and stroke-like lesions in CoQ10 nephropathies [20–22]; ventriculomegaly in patients with *CRB2*-glomerulopathy [23] and subcortical changes in patients with *SGPL1* biallelic pathogenic variants [24] have been described. Ophthalmological examination and hearing evaluation should also be performed in all cases.

2. *What is the preferred time-point for genetic diagnostics?*

We recommend genetic testing as a first choice diagnostic test in every CNS patient. It should be performed as part of the initial patient evaluation and should be considered prior to a renal biopsy.

Once the clinical suspicion of CNS is raised, genetic testing should be initiated. Genetic testing in CNS is a fast, noninvasive and reliable one-time diagnostic measure. Prompt genetic testing has profound effects on clinical decision making as it reduces the time for diagnosis in infants during hospital stay and may enhance the cost-effectiveness of clinical management [25, 26]. Establishing the genetic diagnosis is essential for proper patient management, facilitates the anticipation, and/or swift identification of extrarenal manifestations, informs recurrence risk counseling, and may lead to the identification of genetic defects that may represent phenocopies of nephrotic syndrome [27]. As a general rule, in genetic forms of CNS the use of immunosuppressive drugs should be avoided; instead, appropriate fluid management and proteinuria-lowering RAAS (renin–angiotensin–aldosterone system) blockade at postneonatal age should be introduced promptly to stabilize the patient’s condition and slow renal failure progression (see Boyer et al. in prep. for further details). Particular issues around therapeutic decision making in syndromic forms are discussed further in Question 8. These include but are not limited to (1) being aware of the risk of urogenital tumors and meticulous monitoring of them in *WT1*-associated disease; (2) promptly initiating CoQ10 supplementation in CoQ10-related nephropathies; (3) planning appropriate therapy in GAMOS individuals or subjects with *CRB2* or *SGPL1*-associated disorders; (4) managing endocrine manifestations in individuals with *SGPL1* or *WT1*-related syndromes.

3. *What is the appropriate genetic testing approach?*

We recommend one- or two-step genetic testing depending on the presenting phenotype and financial and/or technical restrictions related to the diagnostics.

**Fig. 1:**
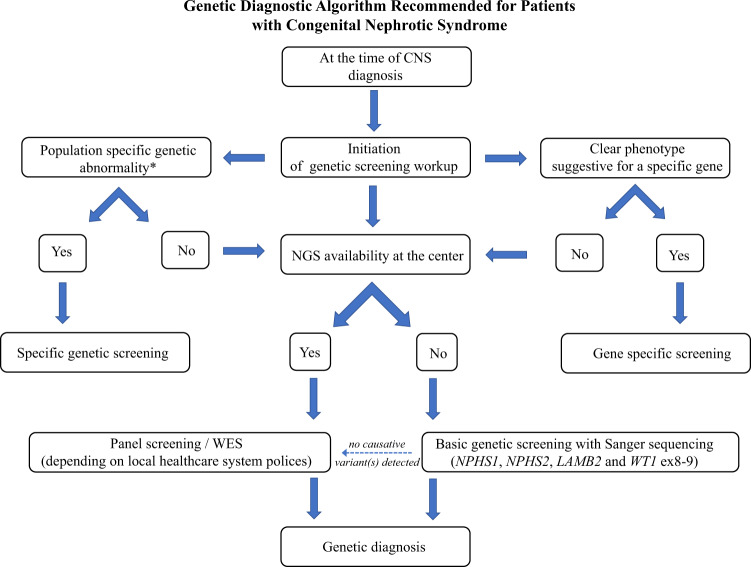
Algorithm for genetic diagnosis in individuals with congenital nephrotic syndrome. Asterisk [*]: applicable for populations where founder mutations have already been well described.

Genetic screening strategies might vary depending on country specific peculiarities including availability and access to genetic testing and reimbursement policy of national healthcare systems (Fig. 1).

In CNS, basic genetic screening of *NPHS1, NPHS2, WT1*, and *LAMB2* genes will uncover underlying genetic abnormalities in >80% of cases. Several other less commonly mutated genes account for an additional ~5% of diagnoses [6, 7, 9, 10, 28, 29]. Due to the wide range of phenotypic variability and genetic heterogeneity of the disease [6, 7, 9, 14, 28, 29] a comprehensive genetic screening comprising all SRNS-related genes is recommended as the first tier method using next generation sequencing technology with either an expanded gene panel or whole exome sequencing (WES). Targeted gene panels have higher depth of coverage of the genes related to a specific phenotype, which allow a higher diagnostic rate. Also, gene panels will not yield incidental findings in genes unrelated with CNS. However, the number of genes that can be examined using a gene panel approach is limited and the covered list of genes should be regularly updated as new genes are continuously identified. Conversely, WES allows detecting variants not only in an established list of known, but also in novel disease-causing genes [30]. However WES has also some important limitations. Complex rearrangements, small copy-number variants and changes within regulatory fragments (promotor, introns, enhancers, silencers etc) might be missed by standard protocols. In particular, WES may have suboptimal coverage of some clinically relevant regions: fragments with high GC content, the mitochondrial genome or duplicated regions (pseudogenes) [31]. These limitations may, to some extent, be overcome by use of hybridization-based custom target enrichment for NGS gene panels.

For patients who present a clear phenotype associated with a syndromic form of CNS, testing the particular related gene can be performed as the first step followed by comprehensive genetic testing if no pathogenic variant is detected. In populations where founder mutations or specific genetic abnormalities have already been well described such as CNS of the Finnish type (*NPHS1* Fin-major and Fin-minor recessive pathogenic variants), the genetic screening strategy may be modified considering population specific features.

Informed consent should be obtained before initiating genetic studies. Informed consent forms should clearly describe the methods that will be applied, and the interpretation and handling of results and incidental findings that would have clinical and psychosocial impacts, for instance male-to-female sex-reversal related to *WT1* dominant pathogenic variants.

4. *Is there a role for karyotyping?*

We recommend karyotype testing to be performed in each CNS patient with ambiguous genitalia and in each phenotypic female with a causative *WT1* mutation.

Establishing chromosomal gender of a patient with CNS with ambiguous genitalia and/or a *WT1* dominant pathogenic variant is necessary for further proper management, endocrine and oncological follow-up in particular [32, 33].

For karyotype testing, a heparin blood sample should be obtained according to local genetic laboratory recommendations.

5. *What kind of samples are needed for genetic testing?*

We recommend performing genetic testing on whole blood (EDTA) or umbilical cord tissue/blood sample.

A blood sample for genetic testing should be obtained as soon as possible as it informs further clinical management of a CNS patient. However, in those patients who have individual blood-sampling limitations or less than 3 months from a blood transfusion, buccal swabs or newborn dried blood spots may also be used [34, 35].

6. *Is there a role for prenatal diagnosis/genetic counseling?*

We recommend prompt genetic counseling in families with a past history or prenatal signs of CNS.

In families with a past history of CNS, recurrence risk counseling by a clinical geneticist/clinical counsellour should be promptly provided. The decision regarding preimplantation genetic diagnosis and prenatal genetic testing should be discussed in light of the local financial, social, and legal settings [36].

7. *What are the phenotype/genotype correlations?*

We suggest to take the following phenotype–genotype information into account for genetic counseling:

### *NPHS1*

The majority of patients in whom CNS manifests in the first week of life will harbor biallelic pathogenic variants in the nephrin gene [10, 14]. The frequency of the disorder is significantly higher in Finland with two *NPHS1* founder mutations (i.e., Fin-major and Fin-minor) in most cases [3–5]. Renal pathology does not exclusively appear as “Finnish type” NS in CNS caused by recessive *NPHS1* pathogenic variants [37]. There is no difference between truncating *NPHS1* variants (i.e., nonsense, frameshift, and splice site) and missense *NPHS1* variants in terms of age at diagnosis, proportion of age at ESKD or death, and proportion of patients who achieved at least transient albumin withdrawal [38]. A subset of patients with CNS and a milder clinical course were described associated with the NM_004646.3: c.3478C>T(p.(Arg1160Ter)) variant [10, 39].

### *PLCE1*

Patients with *PLCE1* (NM_016341.3) splice site recessive pathogenic variants have an earlier age of onset than patients with C-terminal truncating variants (after amino acid residue 1000) or missense variants [6]. *PLCE1* pathogenic variants are mostly associated with diffuse mesangial sclerosis histopathology [6]. Anecdotally, incomplete penetrance has been reported [40, 41].

### *NPHS2*

*NPHS2* (NM_014625.3) biallelic pathogenic variants are the main genetic cause of CNS beginning >1 month after birth [10]. There is no correlation regarding age of onset for truncating versus missense variants [7]. The European founder mutation c.413G>A (p.Arg138Gln) is frequently detected in those CNS patients who do not have *NPHS1*-related disease [10]. Individuals homozygous for the c.413G>A (p.Arg138Gln) variant present NS at a median age of 2 months and generally progress to ESKD after the age of 5 years (median 79 months; range 24–159 months) [10]. Individuals with the c.779T>A (p.(Val260Glu)) variant have an earlier age at onset of nephrotic syndrome and progress more rapidly to ESKD when compared to subjects with the c.413G>A (p.Arg138Gln) variant [10].

The disease-associated allele c.686G>A (p.Arg229Gln) has variant dependent pathogenicity [42, 43]. It is considered pathogenic only when located in *trans* to another recessive pathogenic variant in exon 7 or 8 of *NPHS2*. Such compound heterozygosity generally causes late onset nephrotic syndrome (after the median age of 17 months) [6, 10, 42, 43]. However, a few subjects developed nephrotic syndrome in the first month of life; yet, progressed slowly to ESKD [10].

### *LAMB2*

In *LAMB2* (NM_002292.3), N-terminal truncating recessive pathogenic variants tend to manifest before 2 months of life, whereas C-terminal truncating variants later. Missense variants and small *in frame* deletions have a higher mean age at onset of renal disease and lack of neurologic abnormalities [6]. Diffuse mesangial sclerosis has been identified in 61% of individuals with *LAMB2-*associated NS [44].

### *WT1*

*WT1* dominant pathogenic variants are associated with a wide range of clinical phenotypes that are clearly associated with the type and location of the causative *WT1* variant. More than 90% of the deleterious variants reside in the *hot spot* region (exons 8 and 9 and their intronic junctions) [6, 32, 45, 46].

Classically, individuals with *WT1* dominant pathogenic variants have been subclassified as having Denys–Drash or Frasier syndrome, however these two syndromes may overlap phenotypically to a certain extent. A number of patients present with a milder phenotype that cannot be easily classified as one of these syndromes [32, 45, 46].

Missense substitutions affecting DNA-binding residues are associated with diffuse mesangial sclerosis, early-onset steroid-resistant nephrotic syndrome and rapid progression to ESKD. Truncating variants confer the highest risk of Wilms tumor (~80%) and congenital anomalies of kidney and urinary tact (~25%) but are typically associated with late-onset steroid-resistant nephrotic syndrome [32, 45, 46]. Intronic (KTS) variants usually present as isolated SRNS with the histological picture of FSGS and slow progression to ESKD. Patients with isolated SRNS are genotypic and phenotypic females.

Male-to-female sex reversal (46,XY complete gonadal dysgenesis) occurs exclusively in individuals with intronic KTS dominant pathogenic variants and exonic variants. Urogenital abnormalities have also been described in patients with all types of *WT1* deleterious variants [32, 45, 46].

### CoQ10*-*associated *CNS*

Primary CoQ10 deficiencies that stem from autosomal recessive pathogenic variants in genes involving endogenous CoQ10 biosynthesis can cause nephropathies that are collectively referred to as CoQ10-associated nephropathies. Of them, *COQ2, COQ6,* and *PDSS2* biallelic variants have been shown to be related to CNS. As CoQ10 is essential for mitochondrial electron transport, many organs can be affected, therefore multisystemic involvement is a cardinal feature of these disorders. There are no established genotype–phenotype correlations, potentially due to the small number of patients in the literature. In addition to progressive nephropathy, *COQ2* pathogenic recessive variants typically manifest with signs of progressive encephalopathy (including ataxia, generalized amyotrophy, retinitis pigmentosa, bilateral sensorineural deafness, hypotonia, and psychomotor delay), hypertrophic cardiomyopathy, as well as diabetes [20, 21]. *COQ6* recessive pathogenic variants are associated with severe infantile onset progressive SRNS resulting in end-stage renal failure and sensorineural hearing loss, central nervous system involvement, congenital heart disease, and motor retardation [13, 47]. *PDSS2* recessive pathogenic variants are associated with Leigh syndrome, a progressive and severe neurodegenerative disorder, which may become evident within the first months of life and may result in early death. Affected individuals usually show global developmental delay or developmental regression, hypotonia, ataxia, dystonia, and ophthalmologic abnormalities such as nystagmus or optic atrophy. Basal ganglia and/or brainstem or brain imaging show T2-weighted hyperintensities [22].

It most commonly presents as a progressive and severe neurodegenerative disorder with onset within the first months or years of life, and may result in early death. Affected individuals usually show global developmental delay or developmental regression, hypotonia, ataxia, dystonia, and ophthalmologic abnormalities, such as nystagmus or optic atrophy. The neurologic features are associated with the classic findings of T2-weighted hyperintensities in the basal ganglia and/or brainstem on brain imaging.

### Galloway–Mowat syndrome

GAMOS is a phenotypically heterogeneous disorder characterized by neurodevelopmental defects combined with podocytopathy. Individuals with GAMOS may not present overt dysmorphic features, however pre- or postnatal microcephaly should be considered its hallmark. Central nervous system abnormalities result in severely delayed psychomotor development and propensity to seizures. Additional clinical features include skeletal anomalies and various degrees of growth retardation [18].

There is high inter- and intrafamilial variability concerning renal involvement with regard to age at onset and type of kidney disease; some individuals may not even have renal disease [17, 48].

Recessive pathogenic variants in a number of genes, including *WDR73, LAGE3, OSGEP, TP53RK*, and T*PRKB* encoding subunits of the KEOPS complex [17, 18, 48]; *WDR4* encoding an enzyme required for a specific posttranscriptional modification of tRNA [49]; and *NUP107* and *NUP133* encoding nuclear pore complex proteins [50] have been implicated in the pathogenesis of the disorder. This significant genetic heterogeneity and extreme rarity of the disorder with less than 50 patients described so far hamper precise genotype–phenotype analyses.

### *SGPL1*

Biallelic pathogenic variants in *SGPL1* result in a podocytopathy and primary adrenal insufficiency. Additional features include ichthyosis, acanthosis, immunodeficiency manifesting as lymphopenia, and recurrent bacterial infections. About half had variable neurologic abnormalities including ataxia, cognitive decline, loss of motor skills, impaired speech, and sensorineural hearing loss. There was a significant variability of the extra-adrenal and extrarenal features in the ~30 individuals reported so far [24, 51, 52].

### *CRB2*

Biallelic pathogenic variants in *CRB2* result in a glomerulopathy with additional systemic features in a minority of cases [53]. The more severe disease manifests already prenatally with renal corticomedullary cysts and structural abnormalities of the central nervous system, ventriculomegaly in particular. In some, additional defects of the radius or postaxial polydactyly is also noted. Most affected pregnancies have been terminated [23]. No genotype–phenotype data exist due to the extreme rarity of the disorder.

### Histopathology

Diffuse mesangial sclerosis is associated with dominant pathogenic variants in *WT1* (23.1%) and biallelic pathogenic variants in *PLCE1* (17.8%), *LAMB2* (13.6%), and *NPHS1* (4.9%) [6]. CoQ10-associated nephropathies may be associated with focal and segmental glomerulosclerosis, focal mesangial sclerosis, and collapsing glomerulopathy [20, 54]. Increased and dysmorphic mitochondria in podocytes in electron microscopy are highly suggestive for CoQ10-associated nephropathy. Yet, absence of mitochondrial abnormalities does not exclude CoQ10-associated nephropathy diagnosis.

8. *How to manage syndromic forms?*

In addition to standard clinical management of CNS described in detail elsewhere (Boyer et al. in prep.) we recommend that all syndromic CNS patients should be managed by a multidisciplinary team as described below.

### *NPHS2*

We recommend cardiac evaluation in patients with *NPHS2* biallelic pathogenic variants as cardiac anomalies have been shown in 89% of patients with the c.412C>T(p.(Arg138*)) variant [55]. Despite a few case reports describing partial or complete remission after immunosuppressive treatment, by principle patients with biallelic *NPHS2* pathogenic variants respond neither to standard steroid treatment nor to intensified immunosuppressive treatment [7, 56, 57]. Therefore, we recommend not to use immunosuppresive regimens but to use RAAS blockade in such patients.

### *WT1*

We recommend individuals with *WT1*-glomerulopathy to be evaluated for urogenital malformations. Oncological surveillance for Wilms tumor and gonadoblastoma should be applied. Subjects with exonic variants should be monitored for Wilms tumor with abdominal US performed every 3 months until the age of 7 years [58]. After reaching ESKD, bilateral nephrectomy should be considered to prevent the development of Wilms tumor, in particular in individuals carrying truncating variants [32, 59]. In subjects with a 46,XY karyotype and a female phenotype (i.e., complete gonadal dysgenesis), we recommend bilateral gonadectomy due to increased gonadoblastoma risk [32].

*WT1* patients should be managed by a multidisciplinary team comprising a clinical geneticist, pediatric oncologist for Wilms tumor and gonadoblastoma surveillance, pediatric endocrinologist, pediatric surgeon, and psychologist in cases of disorders of sex development.

### *LAMB2*

We recommend detailed ophthalmological examination in children with *LAMB2* biallelic pathogenic variants, even though individuals with missense pathogenic variants may display variable phenotypes ranging from a milder variant of Pierson syndrome to an isolated CNS. Surviving children may have neurodevelopmental deficits and blindness [44].

Individuals with *LAMB2*-associated glomerulopathy need to be managed by multidisciplinary team composed of pediatric optalmologist, clinical geneticist, pediatric neurologist and rehabilitation team.

### *PLCE1*

We recommend *PLCE1*-related nephropathy to be included in the differential diagnosis in subjects with congenital/infantile nephrotic syndrome associated with diffuse mesangial sclerosis in particular. In general, most individuals are resistant to any immunosuppressive therapy but some selected cases may respond to steroid or cyclosporine treatment [40, 60].

### CoQ10-related mitochondropathies (*COQ2, COQ6,* and *PDSS2* genes)

We recommend performing complete and repeated screening for extrarenal manifestations in patients with biallelic pathogenic variants in *COQ2, COQ6,* and *PDSS2* or presenting phenotype suggestive of CoQ10-related glomerulopathy (hearing deficit, encephalopathy, seizures, ataxia, hypotonia, motor/intelectual disability, elevated lactate levels, and diabetes).

For diagnosis we recommend massive-parallel sequencing of the corresponding genes in the first place. In case of a non-informative genetic result, muscle or skin biopsies may be needed for measuring mitochondrial enzyme activity [61]. Renal biopsy with electron microscopy allows quantitative and qualitative analysis of mitochondria [62].

Individuals with CoQ10-related mitochondropathy need to be managed with a multidisciplinary approach including a pediatric ophthalmologist, audiologist, clinical geneticist, pediatric neurologist, rehabilitation team, and in case of diabetes, pediatric endocrinologist.

We suggest treating the individuals with biallelic pathogenic variants in *COQ2, COQ6,* and *PDSS2* or presenting phenotype suggestive of CoQ10-related glomerulopathy with oral CoQ10 as early as possible.

Few case reports suggest that patients with defective variants in CoQ10 biosynthesis genes treated early with oral CoQ10 have improved outcome [20, 54, 61, 63, 64]. Individuals who respond to treatment exhibit an improvement in proteinuria and sometimes in neuromuscular complaints, however, refractory encephalopathy and seizures have been reported in subjects who had a beneficial effect of CoQ10 treatment on their kidney disease [21]. As the total number of reported patients is low, the exact dose regimen to improve or reverse glomerular damage is unknown. The initial CoQ10 dose applied in several reported cases is 15–30 mg/kg/day divided in three administrations, which might be increased to 50 mg/kg/day [47, 54, 63]. Leukocyte CoQ10 levels can be normal in these patients, and seem not to be helpful for monitoring therapy [54].

### Galloway–Mowat syndrome

We recommend that individuals with GAMOS are managed by a multidisciplinary team including a pediatric nephrologist, pediatric neurologist, clinical geneticist, and physiotherapist. For older children psychotherapeutic, psychological and speech therapy services should be offered. Palliative care may also be considered depending on the severity of the disease.

### *SGPL1*-associated CNS

We recommend patients with *SGPL1*-glomerulopathy to be carefully monitored for adrenal insufficiency. Individuals should be managed by a multidisciplinary team including a pediatric nephrologist, pediatric endocrinologist, pediatric neurologist, clinical geneticist, and physiotherapist. For older children psychotherapeutic, psychological, and speech therapy services should be offered. Palliative care may also be considered depending on the severity of the disease.

9. *What is the inheritance pattern of a hereditary CNS?*

We recommend that each individual with confirmed hereditary CNS will have a genetic consult performed to address the issues of recurrence risk in the family.

The majority of forms of hereditary CNS are inherited in an autosomal recessive manner. That implies a 25% risk of recurrence in subsequent pregancies.

The exceptions are:

- *WT1*—inherited in autosomal dominant manner; the recurrence risk depends whether the genetic defect is familial or occurred de novo (50% vs. <1% due to gonadal mosaicism respectively).
- *LAGE3*—inherited in an X-linked recessive manner; the reccurence risk is 0% for female and 50% for male siblings.

10. *Parents as kidney donors.*

We recommend that parents undergo genetic counseling prior to kidney donation for their child who has CNS with a confirmed genetic diagnosis.

The majority of forms of hereditary CNS are inherited in an autosomal recessive (AR) manner (see #9 for exceptions). This implies that both parents are obligate carriers of one of the defective variants. An extremely rare omission to this rule would be a de novo mutation or misattributed paternity. Carriers of a heterozygous variant in an AR gene can be kidney donors.

However, it cannot be excluded that the parents actually are also homozygotes/compound heterozygotes for the pathogenic variant(s) in the gene associated with a hereditary nephrotic syndrome. *NPHS2*-related SRNS and *WT1*-associated glomerulopathy are the two forms of the disease with the most significant intra- and inter-family variability, with age-dependent penetrance reflecting defective variant type [32, 43]. Moreover, incomplete penetrance has been described in families with a *WT1* pathogenic variant [65].
